# A multi-site feasibility study of a stepped-care telehealth intervention for depression and anxiety in post-treatment cancer survivors at community cancer clinics (WF-30917CD)

**DOI:** 10.1007/s11764-024-01721-0

**Published:** 2025-01-14

**Authors:** Suzanne C. Danhauer, Gretchen A. Brenes, Kathryn E. Weaver, Emily V. Dressler, Grace Westcott, Zhang Zhang, Lingyi Lu, Cheyenne R. Wagi, Rakhee Vaidya, Amarinthia Curtis, Pamala A. Pawloski, Sara Adams, Glenn J. Lesser, Janet A. Tooze

**Affiliations:** 1 Department of Social Sciences & Health Policy, Wake Forest University School of Medicine, Winston-Salem, NC USA; 2 Department of Internal Medicine, Section On Gerontology and Geriatric Medicine, Wake Forest University School of Medicine, Winston-Salem, NC USA; 3 Department of Biostatistics and Data Science, Wake Forest University School of Medicine, Winston-Salem, NC USA; 4 Department of Psychology, Wake Forest University, Winston-Salem, NC USA; 5 Department of Health Policy and Management, Gillings School of Global Public Health, University of North Carolina at Chapel Hill, Chapel Hill, NC USA; 6 Carolina Population Center, University of North Carolina at Chapel Hill, Chapel Hill, NC USA; 7 Department of Hematology and Oncology, Wake Forest University School of Medicine, Winston-Salem, NC USA; 8 Upstate Carolina Community Oncology Research Consortium, Greer, SC 29650 USA; 9 Metro-MN Community Oncology Research Consortium, Minneapolis, MN 55416 USA; 10 Tidelands Health System, Murrells Inlet, SC 29576 USA; 11 Department of Biostatistical Sciences, Wake Forest University School of Medicine, Winston-Salem, NC USA

**Keywords:** Anxiety/depression, Cancer, Cognitive-behavioral therapy, Psychosocial oncology, Survivorship, Telehealth

## Abstract

**Purpose:**

This feasibility study estimated accrual, retention, adherence, and summarized preliminary efficacy data from a stepped-care telehealth intervention for cancer survivors with moderate or severe levels of anxiety and/or depressive symptoms.

**Methods:**

Participants were randomized to intervention or enhanced usual care (stratified by symptom severity). In the intervention group, those with moderate symptoms received a cognitive-behavioral therapy (CBT) workbook/6 bi-weekly check-in calls (low intensity) and severe symptoms received the workbook/12 weekly therapy sessions (high intensity). Depression, anxiety, insomnia, fatigue, quality of life (QOL), fear of recurrence, and cancer-related distress were assessed pre- and post-intervention.

**Results:**

Participants (*N* = 68; ages 36–82; 88% White) were randomized to telehealth (*n* = 34) or enhanced usual care (EUC, *n* = 34), stratified by symptoms (moderate, *n* = 38; severe, *n* = 30). Accrual was 1.8/month with 88% retention and > 75% adherence. For those with moderate symptoms, the low-intensity intervention was associated with better cancer-related distress post-intervention but worse fatigue, insomnia, and physical QOL and and minimal differences for anxiety, depression, fear of recurrence, and mental QOL compared with EUC using clinically meaningful values to assess differences. For those with severe symptoms, the high-intensity intervention was associated with better fatigue, fear of recurrence, cancer-related distress, and physical/mental QOL.

**Conclusions:**

Accrual to a stepped-care telehealth intervention for distressed cancer survivors was lower than expected, but retention and adherence were strong. Data suggest potential impact of the high-intensity intervention.

**Implications for Cancer Survivors:**

A telephone-based CBT intervention where cancer survivors worked with a therapist yielded improvements in fatigue, fear of recurrence, distress, and quality of life.

## Introduction

Psychological concerns are among the most commonly reported unmet needs among post-treatment cancer survivors [1]. Psychosocial distress, including anxiety and depressive symptoms, is common and occurs throughout the survivorship trajectory [2–17]. Approximately half of post-treatment cancer survivors report clinically significant distress [7, 18], which is associated with multiple adverse outcomes, including decreased quality of life (QOL) [12, 19, 20], functional limitations [21, 22], poor sleep [23, 24], increased pain [17], increased healthcare costs [25], and—particularly for those with elevated depressive symptoms—increased mortality [26–28]. Despite these outcomes, psychological symptoms of cancer survivors are often not recognized or treated [29]. Psychosocial interventions for post-treatment cancer survivors may improve mental and physical health, potentially offsetting increased healthcare costs [30–33].

National oncology organizations have responded to this need with guidelines for screening and addressing psychosocial distress [31]. The American Society of Clinical Oncology (ASCO) published guidelines for screening, assessment, and care of emotional distress (anxiety, depression) in adults with cancer [16, 32, 33] that recommend providing all cancer patients with information regarding depression and anxiety and screening for distress throughout the trajectory of care. Cognitive-behavioral therapy (CBT; an evidence-based psychological treatment focused on exercises to modify thoughts/behaviors and enhance coping), among other options, is recommended for patients with moderate and severe depression and anxiety [34–36]. Interventions based on these guidelines are needed, especially those addressing accessibility barriers to psychological care and are suitable for community oncology where a majority of patients receive treatment. Accessibility to psychosocial interventions can increase through telehealth. A recent review suggests that telehealth interventions significantly improve anxiety and depression levels in cancer patients [37].

The present study builds on psychosocial screening/treatment guidelines and our previous trial of telephone-based CBT for older adults with Generalized Anxiety Disorder (NIMH 1R01MH083664; PI: Brenes), which demonstrated high acceptability and efficacy for reducing anxiety and depressive symptoms in a rural geriatric population [38]. While ASCO distress guidelines apply to survivors with all cancer types across the cancer treatment and survivorship continuum, we focused on post-treatment survivors of several cancers that generally have longer survival times (non-metastatic breast, colorectal, prostate, uterine, and cervical cancers and any stage lymphoma). The purpose of this study was to gather preliminary data on an adapted CBT intervention for distressed post-treatment cancer survivors with these cancer types in community oncology practices.

This study was conducted through the Wake Forest National Cancer Institute Community Oncology Research Program Research Base (WF NCORP RB) (UG1CA189824), a national network designed to increase community participation in cancer trials. This feasibility study aimed to: (1) estimate recruitment, accrual, retention, and adherence (primary outcomes) to a stepped-care telehealth intervention versus usual care for post-treatment cancer survivors with moderate or severe levels of emotional distress and (2) obtain preliminary data on efficacy measures, including anxiety, depressive symptoms, insomnia, fatigue, fear of recurrence, cancer-related distress, and health-related quality of life (secondary outcomes).

## Methods

### Study population

Inclusion criteria were as follows: (1) age ≥ 18 years; (2) elevated depression and/or anxiety score [ ≥ 8 on the Patient Health Questionnaire (PHQ-9) [39],  ≥ 10 on the Generalized Anxiety Disorder-7 (GAD-7) [40]]; (3) history of treated breast, colorectal, prostate, gynecologic cancer (Stages I, II, or III), or any stage lymphoma; (4) 6–60 months post-treatment; (5) residence in California, Georgia, Illinois, Kansas, Michigan, Minnesota, Missouri, New Mexico, North Carolina, North Dakota, South Carolina, Virginia, Tennessee, or Wisconsin with a study-trained therapist in each respective state (state requirement due to licensure laws); (6) ability to speak English; and (7) telephone access. The study began with an inclusion criterion of residency in a rural zip code as defined by the Rural–Urban Commuting Areas (RUCA) Version 3.1 codes [41]; this criterion was dropped in January 2021 to increase accruals. Exclusion criteria were as follows: (1) psychotherapy in last 30 days; (2) active alcohol or substance abuse; (3) history of prostate cancer or non-Hodgkin’s lymphoma with only active surveillance; (4) progressive cancer; (5) psychotic symptoms in last 30 days; (6) active suicidal ideation; (7) change in psychotropic medication in last 30 days; (8) hearing loss that would preclude participating in telephone sessions; and (9) inability/unwillingness to provide two emergency contacts.

### Study design

In this randomized, multi-site, feasibility study, participants were randomized to a stepped-care telehealth intervention (tailored to symptom level) or enhanced usual care (EUC) group, stratified by symptom severity. *Moderate distress* was defined as a PHQ-9 score of 8–14 and/or a GAD-7 score of 10–14, and *severe distress* was defined as a PHQ-9 score of 15–27 or a GAD-7 score of 15–21 [39, 40]. If a participant was in the severe category on either the PHQ-9 or the GAD-7, they were considered to be in the severe category; otherwise, they were in the moderate category. Participants completed visits at baseline, Week 7, and Week 13.

### Recruitment

Cancer survivors were recruited through NCORP practices through the WF NCORP RB. The NCI Central IRB approved this study (WF-30917CD). The study was registered with ClinicalTrials.gov (NCT03060096).

### Eligibility criteria

The accrual goal was 90 participants. Screening was completed by a designated research staff member at each NCORP component via a two-step process. First, to minimize participant burden, referred potential participants waived documentation of informed consent and completed the Patient Health Questionnaire-2 (PHQ-2) [42] and a single item (“Feeling nervous, anxious or on edge” over the past two weeks with responses 0 = not at all, 1 = several days, and 2 = more than half the days) from the GAD-2 [43, 44]. If a participant scored ≥ 2 on the PHQ-2 [42] or ≥ 1 on the anxiety item, that person was invited to complete the full study screening and an abbreviated informed consent form to proceed. They then completed the PHQ-9 [39] and GAD-7 [40]. If the participant met all eligibility criteria, staff at sites obtained full informed consent and completed baseline measures. Those reporting no/mild distress were offered written survivorship resources and not enrolled.

### Data collection

Interviewer-administered measures were administered and collected by a research staff person at the NCORP component site. Self-administered measures were completed by mail or by telephone. Measures were completed at baseline, Week 7 (abbreviated version of measures), and at Week 13 (post-intervention). At the end of the intervention, participants were mailed self-administered measures. Participants who did not complete these forms were contacted to complete the measures by telephone.

### Intervention

After completing the baseline visit, participants were randomized into stepped-care telehealth or EUC, stratified by symptom severity. Both telehealth groups received a CBT workbook adapted for cancer survivors that focused on techniques for managing anxiety, depression, and distress. Chapter topics included the cognitive-behavioral model of depression and anxiety, relaxation techniques, cognitive restructuring, problem solving, worry control, exposure, behavioral activation, assertive communication, social support, and relapse prevention, with additional optional chapters (e.g., pain, sleep).

#### Low intensity stepped-care telehealth (hereafter referred to as low-intensity intervention)

For participants with moderate distress, the *low-intensity intervention* consisted of the self-guided CBT workbook and bi-weekly (i.e., every 2 weeks) check-in calls from research staff at NCORP component sites. During the check-in calls (5–10 min), site staff inquired about changes in symptom severity in the past 2 weeks, provided minimal support, and did not offer advice. Participants with a 1 standard deviation increase and a total score of ≥ 15 on either the PHQ-9 [39] or GAD-7 [40] at Week 7 were moved to the high-intensity group.

#### High-intensity stepped-care telehealth (hereafter referred to as high-intensity intervention)

For participants with severe distress, the *high intensity intervention* consisted of a CBT workbook with 12 accompanying weekly telephone sessions. Each week, the participant attended a 45-min telephone session with a study therapist and was assigned one workbook chapter to review and daily exercises to complete. Study therapists met the following requirements: Master’s or doctoral degree in counseling, marriage and family therapy, psychology, or social work; licensed as an independent mental health provider; and experience with CBT.

#### Enhanced Usual Care (EUC)

EUC participants received “Facing Forward: Life after Cancer Treatment,” a book developed by the National Cancer Institute to assist with the transition from active treatment to survivorship [45]. They also received local referrals/resources, including support groups and mental health providers. Upon study completion, they received a copy of the CBT workbook. They did not have routine contact with site staff unless it was needed for a study question or to complete study measures.

### Measures

*Demographic information* included self-reported date of birth, gender, race, ethnicity, marital status, education, and insurance status. In place of asking about income, participants answered yes/no to: “During the past 4 weeks, did you have enough money to meet the daily needs of your family?” *Clinical information* obtained from medical records included cancer type, month/year of diagnosis, cancer stage at time of diagnosis, treatment/date of last treatment, and current medications. Cancer recurrence, additional malignancy diagnosis, and treatment data were collected (if applicable) during the study.

*Feasibility measures* included the following: (1) recruitment rate, number of individuals who met all eligibility criteria and percent who agreed to participate; (2) accrual rate, mean number of participants recruited per month; (3) retention, proportion of participants who completed the Week 13 visit; and (4) adherence, number of therapy (high-intensity intervention) or check-in (low-intensity intervention) sessions completed. The protocol specified a priori that a dropout rate of > 30% (defined as 100% minus percent retained) would indicate a larger study may not be feasible, and the accrual rate would be 8–9 participants/month.

*Patient-reported outcomes (interviewer-administered)* included the following: (1) PHQ-9 [39] (baseline, Week 7, Week 13); (2) GAD-7 [40] (baseline, Week 7, Week 13); and the Cornell Services Index (baseline, Week 13) [46], a structured interview that assesses frequency of medical outpatient visits, psychiatric/psychotherapeutic visits, and intensive services (e.g., hospitalizations, home health visits).

*Patient-reported outcomes (self-reported)* collected at baseline and Week 13 and included the following: (1) the Fear of Recurrence Inventory (FCRI)-Severity Subscale [47] is a 9-item short form of the 42-item FCRI for the brief screening of fear of cancer recurrence. Items are rated from 0 (not at all) to 4 (a great deal) to measure the presence and severity of fear of recurrence. The measure is widely used and has strong reliability and validity (Cronbach’s α = 0.75). (2) The Insomnia Severity Index [48] is a seven-item measure of insomnia symptoms, interference with daily functioning, and related distress caused by sleep issues. It has demonstrated reliability and validity in screening primary care patients for insomnia [49]. (3) The PROMIS–Fatigue Scale–Short Form 8a [50], a measure of the experience and impact of fatigue, was scored using t-scores. (4) The SF–36 Health Survey [51] is a measure of quality of life consisting of 36 items that form 8 subscales combining into two domains: the Physical Component Summary (PCS) and the Mental Component Summary (MCS); scores range from 0 (maximum impairment) to 100 (no impairment). It has demonstrated good internal validity and construct validity. (5) The Impact of Events Scale–Revised (IES-R) [52] is a 22-item self-report measure of cancer-related distress over the past week.

#### Adherence for high-intensity intervention participants

At the end of each session, therapists rated the participant on level of intervention adherence over the past week (1 = not adherent at all, not prepared, did not read, and did not do homework to 5 = extremely adherent, prepared, read the chapter, and did homework).

#### Intervention fidelity

For the high-intensity intervention group, one of the study PIs (GAB) rated 10% of randomly selected therapy sessions (1 session per participant) for therapist adherence to the protocol (0 = no adherence to 8 = optimal adherence) and overall therapist competence (0 = none to 8 = excellent). For the low-intensity intervention group, one of the study PIs (SCD) rated 10% of randomly selected check-in calls for protocol adherence. Adherence was based on three questions (yes/no): Did the staff person inquire about worry and anxiety symptoms? Did the staff person inquire about depressive symptoms? Did the staff person provide any specific coping strategies?

#### Intervention costs

Tracking logs were used to record the printing/shipping costs for the workbook and therapist fees *per participant*. Further, we tracked the training cost *per therapist* for clinical training in the intervention delivery (not for research training costs, e.g., study procedures such as how to complete documents).

### Statistical analysis

The target sample size (N = 90) was based on feasibility, specifically estimating confidence intervals for dropout, recruitment, and accrual within 7.2%, 8.2%, and 8.7%, respectively. Feasibility statistics were summarized using descriptive statistics and 95% confidence intervals. Descriptive statistics of the sample were calculated by symptom severity strata. The descriptive analysis of the implementation costs for this project includes the material costs (booklets and shipping), therapist session costs, and training costs. Per-participant costs were calculated and summarized for both interventions. All comparisons of intervention groups were done using an intent-to-treat framework, including retaining a participant in the low-intensity group for analyses after moving to the high-intensity intervention group, per protocol. Mixed models with a random subject effect were used to model the patient reported outcomes by study visit and intervention group separately by symptom severity strata; contrasts were used to estimate differences and 95% confidence intervals by time point. No formal hypothesis testing was performed. Although the study was not powered to detect significant differences, we compared the point estimates of between-group differences to standards of minimally clinically important differences (MCIDs) to evaluate whether between-group differences met or exceeded the MCID to evaluate meaningfulness of the changes observed.

## Results

### Sample description

See Table 1 for sample characteristics (N = 68). Median age was 64 years (range: 36–82), with 93% of participants being female, 88% White, 7% Black, 4% Native American, and 99% non-Hispanic. The sample was over two-thirds rural (71%). When asked whether they had enough money to meet daily needs, 84% reported yes. The most common cancer site was breast cancer (74%) followed by colorectal (9%), non-Hodgkin lymphoma (7%), uterine cancer (4%), prostate cancer (4%), and Hodgkin lymphoma (2%). Most (82%) had Stage I or II cancers; 91% had undergone surgery, 54% chemotherapy, and 62% radiation therapy. The number of months post cancer treatment ranged from 7 to 55 (mean = 26.5, SD = 15.4). A single participant reported a cancer recurrence at Week 13. A majority of participants (62%) reported taking psychotropic medication at baseline. During the study, 24% on of those on psychotropic medications at baseline reported changes in dose or medications (12% increased dose (N = 5; 1 EUC, 4 low-intensity intervention); 5% decreased dose (N = 2; 1 EUC, 1 low-intensity intervention); 2% (N = 1, EUC) changed psychotropic medications; and 5% (N = 2, EUC) stopped a psychotropic medication); 1 low-intensity intervention participant started a psychotropic medication during the study. Although it was an exclusion criteria that was denied by all participants at screening, a few participants reported outside counseling at baseline on the Cornell Services Index (N = 3; 2 EUC, 1 low-intensity intervention) or during the study (N = 4; 2 EUC, 1 low-intensity intervention, 1 high-intensity intervention).

**Table 1:** Baseline characteristics of study sample (*N* = 68)

		Total *N* (%)	Moderate symptom			Severe symptom		
			All *N* (%)	EUC *N* (%)	Low-intensity *N* (%)	All *N* (%)	EUC *N* (%)	High-intensity *N* (%)
Characteristic	**Category**	**68**	**38**	**19**	**19**	**30**	**15**	**15**
Age (y)	Median (range)	64 (36–82)	65 (36–82)	64 (45–98)	65 (36–82)	61 (40–77)	60 (45–76)	63 (40–77)
Months since last cancer treatment	Mean (SD)	68 (range: 7–55)	26 (15)	25 (15)	27 (15)	27 (16)	26 (16)	28 (17)
Gender	Female	63 (93%)	35 (92%)	17 (90%)	18 (95%)	28 (93%)	14 (93%)	14 (93%)
	Male	4 (6%)	2 (5%)	1 (5%)	1 (5%)	2 (7%)	1 (7%)	1 (7%)
	Other	1 (1%)	1 (3%)	1 (5%)	0 (0%)	0 (0%)	0 (0%)	0 (0%)
Race	Black	5 (7%)	3 (8%)	2 (11%)	1 (5%)	2 (7%)	1 (7%)	1 (7%)
	White	60 (88%)	32 (84%)	16 (84%)	16 (84%)	28 (93%)	14 (93%)	14 (93%)
	American Indian	3 (4%)	3 (8%)	1 (5%)	2 (11%)	0 (0%)	0 (0%)	0 (0%)
Ethnicity	Non-Hispanic	67 (99%)	38 (100%)	19 (100%)	19 (100%)	29 (97%)	14 (93%)	15 (100%)
Marital status	Married	39 (57%)	19 (50%)	8 (42%)	11 (58%)	20 (67%)	11 (73%)	9 (60%)
Education	Less than high school	6 (9%)	2 (5%)	2 (10%)	0 (0%)	4 (13%)	0 (0%)	4 (27%)
	High school	24 (35%)	14 (37%)	7 (37%)	7 (37%)	10 (33%)	7 (47%)	3 (20%)
	Some college	38 (56%)	22 (58%)	10 (53%)	12 (63%)	16 (54%)	8 (53%)	8 (53%)
Rural	Yes	48 (71%)	24 (63%)	12 (63%)	12 (63%)	24 (80%)	12 (80%)	12 (80%)
Ability to pay for basics	Yes	57 (84%)	33 (87%)	15 (79%)	18 (95%)	24 (80%)	14 (93%)	10 (67%)
	No	11 (16%)	5 (13%)	4 (21%)	1 (5%)	6 (20%)	1 (7%)	5 (33%)
Insurance Status	Medicare	28 (41%)	19 (50%)	10 (53%)	9 (47%)	9 (30%)	4 (27%)	5 (33%)
	Medicaid	8 (12%)	2 (5%)	2 (11%)	0 (0%)	6 (20%)	2 (13%)	4 (27%)
	TriCare	3 (4%)	1 (3%)	0 (0%)	1 (5%)	2 (7%)	1 (7%)	1 (7%)
	Private/Other	40 (59%)	23 (61%)	12 (63%)	11 (58%)	17 (57%)	10 (67%)	7 (47%)
Cancer site	Breast	50 (74%)	29 (76%)	16 (84%)	13 (68%)	21 (70%)	10 (67%)	11 (73%)
	Colorectal	6 (9%)	3 (8%)	1 (5%)	2 (11%)	3 (10%)	1 (7%)	2 (13%)
	Non-Hodgkin lymphoma	5 (7%)	2 (5%)	0 (0%)	2 (11%)	3 (10%)	1 (7%)	2 (13%)
	Uterine cancer	3 (4%)	2 (5%)	1 (5%)	1 (5%)	1 (3%)	1 (7%)	0 (0%)
	Prostate cancer	3 (4%)	2 (5%)	1 (5%)	1 (5%)	1 (3%)	1 (7%)	0 (0%)
	Hodgkin lymphoma	1 (2%)	0 (0%)	0 (0%)	0 (0%)	1 (3%)	1 (7%)	0 (0%)
Cancer stage	I	33 (48%)	17 (45%)	11 (58%)	6 (32%)	16 (53%)	6 (40%)	10 (67%)
	II	23 (34%)	13 (34%)	5 (26%)	8 (42%)	10 (33%)	6 (40%)	4 (27%)
	III	8 (12%)	6 (16%)	2 (11%)	4 (21%)	2 (7%)	1 (7%)	1 (7%)
	IV (lymphoma only)	2 (3%)	1 (3%)	0 (0%)	1 (5%)	1 (3%)	1 (7%)	0 (0%)
	Localized (prostate only)	2 (3%)	1 (3%)	1 (5%)	0 (0%)	1 (3%)	1 (7%)	0 (0%)
Cancer treatment	Surgery	62 (91%)	36 (95%)	19 (100%)	17 (90%)	26 (87%)	12 (80%)	14 (93%)
	Chemotherapy	37 (54%)	17 (19%)	7 (37%)	10 (53%)	20 (67%)	10 (67%)	10 (67%)
	Radiation	42 (62%)	27 (71%)	13 (68%)	14 (74%)	15 (50%)	6 (40%)	9 (60%)
On psychotropic medicine	Yes	42 (62%)	24 (63%)	12 (63%)	12 (63%)	18 (60%)	8 (53%)	10 (67%)

### Feasibility

**Fig. 1:**
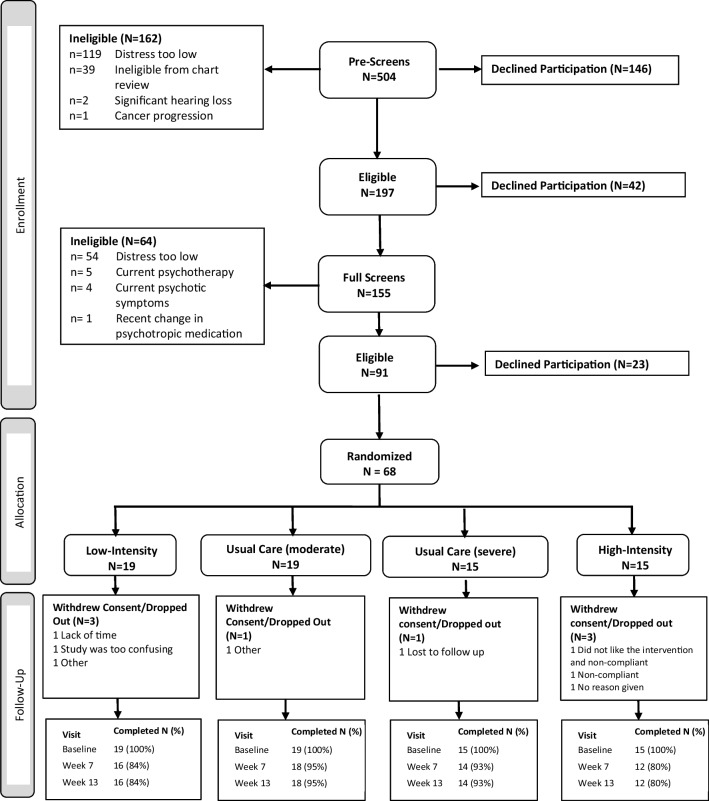
CONSORT diagram

The study was open for *recruitment* from 7/19/18 to 9/17/21 (38 months). The study closed before reaching our goal of 90 patients due to failure to meet accrual targets during the projected recruitment period. In total, 504 patients were screened from 29 NCORP practices (Fig. 1). Of those, 197 proceeded to the full screen (Fig. 1), and 42 declined participation. Of those who proceeded to the full screen, 91/155 were eligible to participate. Twenty-three declined enrollment after the full screen (recruitment rate 75%, 95% CI: 66%, 84%). The remaining 68 enrolled in the study (13% of all screened potential participants).

The *accrual* rate was 1.8 participants/month (95% CI, 1.4, 2.3). With 60 of 68 participants completing the final study visit, the overall *retention* was 88% (95% CI: 78%, 95%; 80% for the high-intensity intervention; 84% for the low-intensity intervention; and 94% for EUC).

Regarding *adherence*, the high-intensity intervention participants completed an average of 9.4 of 12 sessions. One high-intensity intervention participant completed zero sessions. For those who successfully completed the study in the high-intensity intervention group, 95% of sessions (SD = 14%) were completed, on average. On average, the high-intensity intervention group completed 4.5 (SD = 2.9) homework exercises before each therapy session. Therapist-rated participant adherence (rating from 0 to 5) was 3.9 (SD = 1.2).

The low-intensity intervention group participants completed an average of 4.9 of 6 bi-weekly check-in calls. Two low-intensity intervention participants completed 0 check-in calls. Per protocol, one low-intensity intervention participant was moved to the high-intensity intervention group due to increased distress symptoms at Week 7. For those who successfully completed the study in the low-intensity intervention group, 91% of sessions were completed (SD = 20%).

### Intervention fidelity

For the low-intensity intervention group, 100% of the randomly selected check-in calls were adherent to the protocol. For the high-intensity intervention group, the average therapist adherence rating was 7 (SD = 1.1), and competence rating was 7.3 (SD = 0.8) (possible scores ranged from 0 to 8).

### Differences in patient-reported outcomes

Results by symptom strata, intervention group, and time (summarized in Table 2) indicated that Week 13 differences between groups for the *moderate symptom strata* (i.e., low-intensity intervention v. EUC) were small for anxiety, depression, fear of recurrence, and mental quality of life. PROMIS fatigue score was approximately 4 points higher in the intervention v. EUC group, primarily driven by a decline in the EUC group. For fatigue (PROMIS Fatigue Scale Short Form 8a), a MCID of 4 points is considered clinically meaningful [53]. Insomnia was 3.8 points higher in the intervention v. EUC group. While this difference did not reach the MCID (6 points) for the Insomnia Severity Index, confidence intervals included the MCID. At Week 13, the intervention group also had a lower physical quality of life score of 4.6 points compared with the EUC. This difference was close to being meaningfully lower in the intervention group in the moderate strata (MCID of 5), and the 95% CI included the MCID. For cancer-related distress, the difference between groups for the moderate strata (− 0.2 points) was meaningful (MCID = 0.2 points) [54].

**Table 2:** Preliminary data on efficacy and variability of stepped-care telehealth vs. enhanced usual care

		Moderate symptoms								Severe symptoms						
Measure	Time point	Enhanced usual care			Stepped care low intensity				Difference in LS mean (95% CI)	Enhanced usual care			Stepped care high intensity			Difference in LS mean (95% CI)
		N	LS mean	SE	N	LS mean		SE		N	LS mean	SE	N	LS mean	SE	
Anxiety (GAD-7)^a^	Baseline	19	8.2	0.8	19	8.5	0.8		—	15	15.6	1.3	15	15.9	1.3	—
	Week 7	18	5.0	0.8	16	5.8	0.8		0.8 (− 1.5, 3.1)	14	13.4	1.3	12	8.8	1.4	− 4.7 (− 8.6, − 0.8)
	Week 13	18	5.9	0.8	16	4.4	0.8		− 1.5 (− 3.8, 0.8)	14	9.5	1.4	12	8.0	1.5	− 1.5 (− 5.6, 2.6)
Depression (PHQ-9)^b^	Baseline	19	9.4	0.8	19	10.5	0.8		—	15	14.9	1.1	15	17.4	1.1	—
	Week 7	18	6.5	0.8	16	6.9	0.9		0.4 (− 1.9, 2.8)	14	13.7	1.2	12	10.3	1.3	− 3.5 (− 7.0, 0.1)
	Week 13	18	6.8	0.8	16	6.4	0.9		− 0.4 (− 2.8, 2.0)	14	11.2	1.3	12	9.5	1.3	− 1.7 (− 5.4, 2.0)
Fatigue (PROMIS Fatigue)^c^	Baseline	19	58.7	1.8	19	59.0	1.8		—	15	66.7	2.1	15	64.8	2.1	—
	Week 13	18	54.2	1.9	16	58.2	2.1		4.0 (− 1.8, 9.7)	14	65.2	2.2	12	58.2	2.5	− 7.0 (− 13.9, − 0.1)
Fear of Recurrence (Fear of Recurrence Inventory)^d^	Baseline	19	20.1	1.9	19	21.4	1.9		—	15	26.3	1.8	15	24.3	1.8	—
	Week 13	18	17.9	2.0	16	19.4	2.1		1.5 (− 4.4, 7.4)	14	21.9	1.9	12	16.8	2.1	− 5.0 (− 10.9, 0.8)
Cancer-Related Distress (Impact of Events Scale)^e^	Baseline	19	3.4	0.5	19	4.0	0.5		—	15	5.3	0.6	15	5.4	0.6	—
	Week 13	18	2.9	0.5	16	2.8	0.6		− 0.2 (− 1.7, 1.4)	14	4.5	0.6	12	2.7	0.7	− 1.8 (− 3.6, 0.1)
Insomnia (Insomnia Severity Index)^f^	Baseline	19	12.1	1.3	19	14.0	1.3		—	15	15.9	1.9	15	18.6	1.9	—
	Week 13	18	9.2	1.3	16	12.9	1.4		3.8 (− 0.2, 7.7)	14	13.1	2.0	12	11.4	2.2	− 1.7 (− 7.8, 4.4)
Healthcare Utilization (Cornell Services Index)^g^	Baseline	19	2.5	0.2	19	2.8	0.2		—	15	1.8	0.3	15	2.5	0.3	—
	Week 13	18	2.1	0.2	16	2.5	0.3		0.4 (− 0.3, 1.1)	14	1.8	0.4	12	2.5	0.4	0.8 (− 0.4, 1.9)
Quality of Life – Physical (SF36 Physical Health Composite)^h^	Baseline	19	41.0	2.6	19	40.9	2.6			15	36.7	2.4	15.0	38.6	2.5	—
	Week 13	15	42.2	2.8	16	37.6	3.1		− 4.6 (− 13.1, 3.9)	14	36.9	2.5	12.0	44.3	2.8	7.4 (− 0.4, 15.2)
Quality of Life – Mental (SF36 Mental Health Composite)^h^	Baseline	19	41.3	2.3	19	37.4	2.3			15	29.5	2.7	15.0	28.6	2.8	—
	Week 13	15	44.8	2.5	16	46.6	2.7		1.8 (− 5.7, 9.3)	14	35.9	2.8	12.0	42.0	3.2	6.1 (− 2.7, 14.8)

^a^GAD-7: higher scores = greater anxiety [40]

^b^PHQ-9: higher scores = greater depression [39]

^c^PROMIS Fatigue: higher scores = greater fatigue [50]

^d^Fear of Recurrence Inventory: higher scores = more frequent/severe thoughts of recurrence [47]

^e^Impact of Event Scale: higher scores = greater cancer-related distress [52]

^f^Insomnia Severity Index: higher scores = greater insomnia [48]

^g^Cornell Services Index: higher scores = greater degree of utilization of health services and resources[46]

^h^SF-36 Physical & Mental Composites: higher scores = better quality of life (51)

For the *severe symptom strata* (i.e., high-intensity intervention v. EUC), there were minimal Week 13 differences between the two groups for anxiety and depression. In the intervention group compared with EUC, PROMIS fatigue score was 7 points lower, fear of recurrence was 5 points lower, cancer-related distress was 1.8 points lower, physical quality of life was 7.4 points higher, and mental quality of life was 6.1 points higher. Differences between groups did not approach the MCIDs for the GAD-7 [55] (MCID = 4 points) or for the PHQ-9 [56] (MCID = 5 points), although confidence intervals included these MCID values.

### Intervention costs

Intervention costs included intervention booklets (printing, shipping) and therapist costs (intervention training, therapy fees). We provided intervention participants (low and high intensity) with workbooks. The workbook cost *per participant* was $10 to print and $38 to ship (average cost for 2-day FedEx shipping) for a per-participant workbook total of $48. For the high-intensity intervention, we paid therapists a *per participant* flat fee of $700 regardless of number of sessions. For low-intensity intervention participants, the workbook cost was $48. For high-intensity intervention participants, the workbook cost was $48 plus therapist fees of $700 for a total of $748. In addition, the cost *per therapist* for intervention training was $500 (did not include research-specific training, simply intervention delivery).

## Discussion

This pilot RCT feasibility study did not meet accrual targets for either total number or accrual rate. Several factors may have contributed to lower-than-anticipated accruals. First, sites reported challenges incorporating the multi-step screening process into their workflows. While we tried to imitate a screening scenario appropriate for a clinic setting with the goal of minimizing burden (e.g., initial screening followed by more in-depth screening of “at-risk” patients), the need to consent patients at both steps and potential delay between screenings led to attrition (45% of patients withdrew during screening). For future studies, offering an efficient, single-step screening process would be helpful, such as screening through the medical record patient portal [57]. Second, restrictions against providing therapy across state lines required us to have a study therapist in each state where we enrolled participants. This requirement restricted enrollment to certain states, and we experienced several challenges related to having a therapist available in all states throughout the study. We suggest using the PHQ-8 [58] to determine eligibility given that the PHQ-9 [39] contains a suicide item; sites perceived inclusion of this item a barrier to participation. Third, recruitment may be improved by maintaining broad inclusion criteria. This study was initially focused on post-treatment cancer survivors from rural areas, but this criterion limited site involvement too greatly, so it was eliminated midway through the study. Although we did not meet our accrual targets, we enrolled 75% of eligible participants and retained 88% for 13 weeks. Further, intervention fidelity was high, and intervention adherence exceeded 75%. These results suggest that once participants completed the screening process, they were likely to agree to participate, and, once enrolled, they were engaged, indicating success of intervention implementation. It is anticipated that a more streamlined screening process in a larger study would lead to demonstrated study feasibility.

Our comparison of between-group differences to MCID metrics from the published literature suggests for the moderate symptom group, low-intensity intervention (CBT workbook) v. EUC, no clinically meaningful group differences were seen for most mental health outcomes. In fact, physical health outcomes looked *worse* in the intervention group. Only cancer-related distress appeared to be slightly better in the intervention group. Overall, for physical measures (fatigue, insomnia, physical quality of life), the low-intensity intervention suggests no beneficial effect and possibly harmful effects. Perhaps simply working through a CBT workbook without therapist guidance is unhelpful for those needing additional support.

For the severe symptom group, the comparison of between-group differences examination to MCID metrics suggests that there were no clinically meaningful differences for depression or anxiety between the high-intensity intervention (CBT workbook + weekly therapy sessions) v. EUC. Clinically meaningful differences favoring the intervention group were seen for cancer-related distress, mental quality of life, fatigue, insomnia, and physical quality of life. Given these suggestive findings, the high-intensity intervention warrants further study in distressed post-treatment cancer survivors.

It is notable that this sample of cancer survivors, particularly the severe strata, had high levels of fatigue, with baseline scores approximately 1.5 SD higher than the population average (mean of 50, SD of 10) [50], and similarly low physical QOL scores far below the population average. These levels suggest that an intervention that could lead to improvements in fatigue and physical QOL could be highly impactful for this survivor group. It is also of interest that in the severe strata, we saw clinically meaningful impact for the cancer-specific measures (fear of recurrence, cancer-related distress) and fatigue, a prevalent condition in cancer survivors. Greater clinically meaningful impact in these measures salient to cancer survivors may reflect the tailoring of the workbook for the cancer survivor population. It is not clear how the CBT workbook would have specifically impacted physical and cancer-specific outcomes as it did not contain discussions of physical interventions (e.g., physical activity); while it contained materials specific to sleep and pain, those chapters were optional and likely completed only by participants experiencing such symptoms.

Clear limitations were the small sample size, difficulty with accrual, lack of longitudinal follow-up data, lack of participant feedback on the intervention, and limited potential generalizability of the sample dominated by white female breast cancer survivors. The high percentage of breast cancer survivors compared with other cancer types could have reflected the subspecialty of referring physicians or perhaps a survivor group that may be highly motivated to participate in psychosocial intervention studies. We have found that breast cancer survivors are especially engaged in the NCORP network and many of our studies open to survivors of multiple cancer types recruit predominantly breast cancer survivors [59]. It is possible that the impact of having a heterogeneous group of participants with anxiety, depression, or both could impact the results. It is also possible that starting new psychotropic medications or starting psychotherapy could impact our findings; however, only about one-quarter had changes in medication use, most of whom were in the moderate strata, and only four reported outside counseling during the study, so medication use or outside psychotherapy did not appear to impact the majority of participants. In addition, with the exception of mental QOL, impacts of the intervention in the severe strata tended to be for symptoms that are prevalent in cancer survivors. With our small sample size, however, we are limited in performing subgroup analysis or testing between-group differences. Thus, conclusions about intervention efficacy cannot be made. These limitations notwithstanding, this study has a number of strengths. First, participants were recruited from community-based cancer centers across the country. Another strength is the inclusion of a usual-care control group, so the impact of the stepped-care intervention could be more practically assessed. Additionally, the intervention aligns with ASCO’s 2023 guideline update in which experts found CBT and related therapies have the strongest empirical support and should be used as first-line treatments for managing anxiety and depression in cancer survivors [33].

Future research should consider strategies for identifying potentially eligible post-treatment cancer survivors that minimize staff burden. It may be possible to use routine distress screening or other clinic-collected patient-reported outcomes to identify those most likely to report significant anxiety and/or depression. To address the concern of this study only being available to English speakers, our research team recently completed a similar feasibility study with Hispanic post-treatment cancer survivors via a culturally and linguistically adapted CBT intervention (Danhauer et al., under review) [60]. Future research should seek to similarly expand the populations reached.

## Data Availability

Data are available upon reasonable request to the Wake Forest NCORP Research Base. Please contact ncorp@wakehealth.edu for access and more information.
